# A new SIRT1 inhibitor, MHY2245, induces autophagy and inhibits energy metabolism via PKM2/mTOR pathway in human ovarian cancer cells

**DOI:** 10.7150/ijbs.44343

**Published:** 2020-04-06

**Authors:** In Hwan Tae, Ji Yeon Son, Su Hyun Lee, Mi-Young Ahn, Kyungsil Yoon, Sungpil Yoon, Hyung Ryong Moon, Hyung Sik Kim

**Affiliations:** 1 School of Pharmacy, Sungkyunkwan University, 2066, Seobu-ro, Suwon 16419, Republic of Korea.; 2 Major in Pharmaceutical Engineering, Division of Bio-industry, College of Medical and Life Sciences, Silla University, Busan 46958, Republic of Korea.; 3 Comparative Biomedicine Research Branch, Division of Translational Science, National Cancer Center, 323 Ilsandong-gu, Goyang-si, Gyeonggi-do, 10408, Republic of Korea; 4 College of Pharmacy, Pusan National University, Busandaehak-ro 63, Geumjeong-gu, Busan 46241, Republic of Korea.

**Keywords:** Sirtuin, MHY2245, autophagy, PKM2/mTOR, ovarian cancer

## Abstract

Ovarian cancer is a common gynecological cancer that is found worldwide. Class III histone deacetylase (HDAC) inhibitors, a new class of anticancer agents, induce autophagy in various human cancer cells. The aim of the present study was to investigate the antitumor activity of MHY2245, a new synthetic SIRT inhibitor, on human ovarian cancer cells. We found that MHY2245 exhibited potent cytotoxicity to SKOV3 cells in a time- and concentration-dependent manner. The cytotoxicity of MHY2245 (IC_50_=0.32 µM) was higher than that of doxorubicin (DOX, IC_50_=1.38µM) against SKOV3 cells. MHY2245 significantly inhibited SIRT1 enzyme activity, reduced the expression of SIRT1, increased cell cycle arrest at G_2_/M phase, and induced apoptotic cell death in SKOV3 cells via expression of cytochrome c, cleaved-PARP, cleaved caspase-3, and Bax. This might be associated with blocking of the pyruvate kinase M2 (PKM2)/mTOR pathway. MHY2245 also inhibited tumor growth and reduced tumor size when SKOV3 cells were transplanted into nude mice. Our results indicate that MHY2245 exerts antitumor activity against ovarian cancer cells by blocking the PKM2/mTOR pathway. We suggest that MHY2245 is a promising anticancer agent that disrupts ovarian cancer cell metabolism.

## Introduction

Ovarian cancer accounts for an estimated 239,000 new cases and 152,000 deaths worldwide annually 1-3. Furthermore, ovarian cancer has no specific symptoms until it is late-stage and is more difficult to cure even after radical surgery 4, accounting for a low (26%) 5-year survival rate. Epidemiological studies indicate that hormones and reproductive factors play important roles in the pathogenesis of ovarian cancer 3-5. In addition, previous studies postulated that chemicals in the environment, including endocrine disruptors, also interfere with the actions of steroid sex hormones, and contribute to the development of ovarian cancer 6,7.

Although diagnostic techniques for ovarian cancer have become more refined, reliable chemotherapeutic drugs are still very few. In particular, the advanced stage of an ovarian tumor is difficult to treat by estrogen-deprivation and is associated with increased risk of osteoporotic fragility fractures 5,8. Taken together, over 80% of patients with advanced-stage ovarian cancer have relapses after initial responsiveness to chemotherapeutic drugs 9. As a result, there is a desperate need for novel therapies, particularly those that can mitigate or overcome the resistance to chemotherapy. Therefore, for therapy to be effective against ovarian cancer, it needs to reduce serious adverse reactions and allow a better quality of life.

To date, promising new target strategies to regulate energy metabolism in cancer cells have tried for effective and safe treatment of ovarian cancer 10,11. New modeling tools allow cancer researchers and drug designers to evaluate major intracellular pathways simultaneously, to find novel targets for cancer therapy. Increasing evidence suggests that sirtuin 1 (SIRT1), a class III histone deacetylase (HDAC), plays critical roles in multiple aspects of cancer drug resistance 12. SIRT1 is over-expressed in a majority of ovarian cancers 13,14, implying a role of SIRTs in ovarian tumorigenesis. HighexpressionofSIRT1isalso associated with malignant transformation of human ovarian tissue 15 and with ovarian carcinoma with poor prognosis 16. Therefore, SIRT1 inhibition appears to be a good strategy to overcome cancer drug resistance and improve therapy. However, the mechanisms by which SIRTs inhibitors exhibit anticancer activity through blocking of cancer cells metabolism pathway have raised several questions that need to be addressed. It has been suggested that a decline in energy metabolism may play an important role in activating the cell death pathway and dysregulation of pyruvate kinase M2 (PKM2), which favor both carcinogenesis and the Warburg effect 17. Previous study demonstrated that SIRT1 overexpression is associated with poor outcome and chemo-resistance in ovarian cancer of epithelial origin over-expression, and one of the mechanisms may involve the dysregulation of energy metabolism and stress response 16,18.

In the present study, we synthesized a novel SIRT inhibitor, MHY2245, and investigated its anticancer activity and its relevance in the induction of autophagy in ovarian cancer cells. We found that MHY2245 exerts its effects against ovarian cancer cells through thePKM2/mTOR signaling pathway. Our results indicated a cellular mechanism and therapeutic target of MHY2245 for treatment of ovarian cancer.

## Materials and Methods

### Reagents and antibodies

Doxorubicin hydrochloride (DOX), thiamine hydrochloride,4,6-diamidino-2-phenylindole (DAPI) and propidium iodide (PI) solutions were purchased from Sigma-Aldrich (St. Louis, MO, USA). Dulbecco's modified Eagle's medium (DMEM), penicillin/streptomycin, and 3-(4,5-dimethylthiazol-2-yl)-2,5-diphenyltetrazolium bromide (MTT) were obtained from Thermo Fisher Scientific, Inc. (MA, USA). Fetal bovine serum (FBS) was supplied by GIBCO (NY, USA). An annexin V detection kit was purchased from BD Biosciences, Inc. (NJ, USA). DL-Lactic acid was purchased from Tokyo Chemical Industry Co, Ltd. (Tokyo, Japan). Primary antibodies against cyclin B1, cyclin D1, cell division cycle protein 2 (Cdc2), phospho-Cdc2 (p-Cdc2), cyclin-dependent kinase 4 (CDK4), B-cell lymphoma 2 (Bcl-2), Bcl-2-associated X protein (BAX), cleaved-caspase 3 (clev-cas. 3), cleaved-caspase 6 (clev-cas. 6), cleaved-caspase 9 (clev-cas. 9), cleaved-Poly (ADP-ribose) polymerase (clev-PARP), cytochrome c (Cyt. C), p53, mammalian target of rapamycin (mTOR), phospho-mTOR (p-mTOR), phosphatase and tensin homolog (PTEN), phospho-PTEN (p-PTEN), mouse double minute 2 homolog (MDM2), phospho-MDM2 (p-MDM2), phospho-protein kinase B (p-Akt), pyruvate kinase isozymes M2 (PKM2), autophagy-related gene 5 (Atg5), p21, gamma H2A histone family member X (γH2AX) and Ki-67 were purchased from Cell Signaling (Mt, USA). Primary antibodies against acetyl-p53, phosphoinositide 3-kinases (PI3K), phospho-PI3K (p-PI3K), glucose-6 phosphate (G6P), glucose transporter 1 (GLUT1), hypoxia-inducible factor 1-alpha (HIF-1α), heme oxygenase-1 (HO-1), microtubule-associated proteins 1A/1B light chain 3B (LC3B), nucleoporin p62 (p62), nuclear factor erythroid 2-related factor 2 (Nrf2), Beclin1, SIRT1, SIRT2, SIRT3, SIRT4 and SIRT6 were purchased from Abcam plc. (Cambridge, UK). Primary antibodies against phospho-p21 (p-p21) and β-actin were purchased from Santa Cruz Biotechnology, Inc. (TX, USA). MHY2245 and DOX were dissolved in dimethyl sulfoxide (DMSO) and stored at -20°C until use. These agents were diluted to appropriate concentrations with culture medium containing 1% FBS. The final concentration of DMSO was less than 0.1% (vol/vol) and was present in the corresponding controls.

### Cell lines and culture conditions

The human ovarian cancer SKOV3 (ATCC®HTB-77TM) and OVCAR3 (ATCC®HTB-161), Ishikawa (human endometrial adeno-carcinoma, ECACC 99040201), MCF-7 (human breast adeno-carcinoma, ATCC®HTB-22), DU145 (human prostate carcinoma, ATCC®HTB-81), CAKI-1 (human kidney carcinoma, ATCC®HTB-46) and HK-2 (human kidney normal tubular cell, ATCC®CRL-2190TM) cells were cultured in DMEM supplemented with heat inactivated 10% FBS containing penicillin/streptomycin at 37℃ in 5 % CO_2_ humidified incubator. Culture media were changed every 2-3 days and sub-cultured when the cell population density reached 70%-80% confluence. Cells were seeded at an appropriate density according to each experiment.

### Assay of total HDAC and SIRT1 enzyme activity

Histone deacetylase (HDAC) enzyme activity was measured using fluorogenic HDAC1 assay kits (Enzo life science, Inc. NY, USA), according to the manufacturer's instructions. Briefly, HDAC1 enzyme was incubated with trichostatin A, MHY2245, or DOX at 37 °C for 30 min in the presence of HDAC1 substrates tagged with suitable fluorescent labels. The HDAC1 assay developer (which produces a fluorophore in the reaction mixture) was added and the fluorescence was measured after the reaction was stopped by using a VICTOR3 multilabel plate reader (PerkinElmer, Waltham, MA, USA) at appropriate wavelengths. Enzyme activities were calculated by using GraphPad Prism v7 (San Diego, CA, USA). SIRT1 enzymatic activity was assessed by using commercial kits (ab156065) from Abcam plc. (Cambridge, UK) in accordance with the manufacturer's instructions. First, assay buffer (50 mM TRIS-HCl, pH 8.0, 137 mM sodium chloride, 2.7 mM potassium chloride, 1 mM magnesium chloride, 1 mg/mL bovine serum albumin), SIRT1 enzyme, and either solvent (DMF) or different concentrations of drugs (Suramin, MHY2245, or DOX dissolved in DMF) were mixed with the substrate (p53) and co-substrate (NAD^+^) for 45 min. Thereafter, the stop/developing solutions, containing a mixture of a developer, were added to the microplate and incubated for 30 min at 25°C. The deacetylated peptide reacts with the developer and releases a fluorophore. The fluorophores in both assays were analyzed at an excitation wavelength of 350 nm and an emission wavelength of 450 nm. The inhibitory percentage of the samples on theSIRT1 enzyme activity was calculated as the ratio of fluorescent intensity between samples and vehicle control.

### Cytotoxicity assay

Cell viability was determined by MTT assay, as previously described 19. Briefly, cells seeded in 96-well plates (5 x 10^3^ cells per well) were treated with various concentrations of MHY2245 or doxorubicin for 24 or 48 h. MTT (Final concentration, 1 mg/mL) was added to each well and incubated for 3.5 h. The medium was discarded and 100 μL of DMSO was added to each well, and incubated for 20 min. The absorbance per well was measured at 540 nm using the VERSA Max Microplate Reader (Molecular Devices Corp., CA, USA). The 50% inhibitory concentration (IC_50_) was indicates the drug concentration required for 50% inhibition of control cell viability.

### Colony forming assay

To perform the colony formation assay, SKOV3 (300 cells/plate), OVCAR3 (500 cells/plate), and HK-2 (300 cells per well) cells were seeded respectively in a 6-well plates. To perform colony formation assay, cells (300 cells per well) were seeded in a 6-well plates. After 3 days incubation, cells were treated with either MHY2245 (0.03, 0.1 and 0.3 μM ) or DOX (1 μM ) for 24 h. Colony formation was allowed for 14 days and the fresh medium was changed every 3 days. Colonies were fixed with methanol and stained with Giemsa dye for 10 min at room temperature and rinsed with tap water. Colonies of more than 50 cells counted under a microscope.

### Immunofluorescence assay

SKOV3 cells were plated on 35mm confocal dishes (SPL Life Sciences Co., Pocheon, Korea) and incubated for 24 h until they reached 70% confluency. The cells were then incubated in the presence or absence of MHY2245 (0.03, 0.1 and 0.3 μM) or DOX (1 μM) for 48 h, washed twice with PBS, and fixed in a 4% formaldehyde solution for 10 min at RT. They were permeabilized with 0.1% Triton-X 100 for 10 min, blocked in 1.5% horse serum in PBS for 30 min at RT, and then incubated overnight at 4ºC with primary antibodies in a humidified chamber. After washing three times with PBS, the cells were incubated with anti-rabbit IgG H and L secondary antibody (1:200; Alexa Fluour®488, Abcam plc.) for 1 h. The cells were stained with DAPI solution for 5 min. The dishes were then washed three times with PBS before observing and capturing the images under a fluorescent microscope (K1-Fluo, Nanoscope Systems®, Daejeon, Korea).

### Western blot analysis

Cells were treated with either MHY2245 (0.03, 0.1 and 0.3 µM) or Doxorubicin (DOX) (1 µM) for 48 h and harvested by trypsinization and were washed twice with cold PBS. For isolation of total proteins, cells were suspended in PRO-PREP^TM^ protein extract solution and placed on ice for 30 min. The supernatant was collected after centrifugation at 10,000 ×*g* for 15 min at 4 °C. To isolate the cytosolic and nuclear proteins separately, cells were suspended in 50 mL of lysis buffer I (10 mM HEPES, pH 7.9, 1.5 mM MgCl_2_, 10 mM KCl, 0.5 mM DTT and 0.5 mM PMSF) and placed on ice for 20 min. The supernatant was removed after centrifugation at 12,000 ×*g* for 10 min. The pellet was suspended in 30 mL of lysis buffer II (10 mM HEPES, pH 7.9, 1.5 mM MgCl_2_, 10 mM KCl, 0.5 mM DTT, 0.5 mM PMSF and 0.5% NP-40) and placed on ice for 20 min. The cells were lysed by gentle vortexing, and the nuclei were separated from the cytosol by centrifugation at 12,000 ×*g* for 10 min. The nuclei were suspended in 40 mL of buffer III (5 mM HEPES, pH 7.9, 300 mM NaCl, 1.5 mM MgCl_2_, 0.2 mM EDTA, 0.5 mM DTT, 0.5 mM PMSF and 26% glycerol) and placed on ice and shaken for 30 min. The nuclear proteins were obtained by centrifugation at 12,000 ×*g* for 30 min and stored at -70 °C. Protein concentrations were measured using a protein assay kit (Bio-Rad, Hercules, CA, USA) according to the manufacturer's instructions. Samples, each with 20-30 µg protein, were electrophoresed on 6%-15% SDS PAGE, and transferred to PVDF membranes (Millipore, Billerica, MA, USA). After incubating for 1 h in TNA (10 mM Tris-Cl, pH 7.6, 100 mM NaCl, and 0.5% Tween 20) buffer containing 5% skim milk, the membranes were transferred to relevant primary antibodies (diluted to 1:1000) and incubated overnight at 4℃. This was followed by washing for 1 h with TNT buffer, and then incubation with horseradish peroxidase-conjugated anti-mouse or anti-rabbit antibodies (1:10000, Santa Cruz, CA, USA) for 30 min at room temperature. Fluorescence signals were developed using an enhanced chemiluminescence (ECL)-plus kit (Amersham Biosciences, Amersham Buckinghamshire, UK). The band intensities were quantified using Image-J software (NIH, Bethesda, MD) and normalized using the expression level of β-actin.

### Cell cycle analysis

Cell cycle perturbations were studied using flow cytometry to measure the proportion of cells in different phases of the cell cycle. For this, the cells were treated with different concentrations of MHY2245 (0.03, 0.1, or 0.3 μM) for 48 h. The total number of cells, including the ones in suspension and those adhering to the walls, were harvested separately for different cell cycle stages, and washed in 1% bovine serum albumin (BSA) before fixing in 95% ice-cold ethanol containing 0.5% Tween-20 at -20 °C for 1 h. These cells (1 x 10^6^) were again washed in 1% BSA, stained with cold propidium iodide (PI) staining solution (10 μg/mL PI and 100 μg/mL RNase in PBS) in the dark for 30 min at room temperature. Cell cycle profiles were obtained using a GuavaReasyCyte flow cytometer (Merck Millipore, Inc., Mt, USA). Debris and aggregates were gated out during data acquisition and 5000-10,000 events were collected from each sample. Data were analyzed with the Cell Quest Pro software.

### DAPI staining

Morphological changes in the nuclear chromatin of the apoptotic cells were identified by staining with DAPI. Cells were grown in 6-well plates at a density of 1 x 10^5^ cells per well for 48 h before treating with relevant drugs for 48 h. They were then washed with cold PBS, fixed with methanol for 30 min, rewashed and stained with 200 mL of DAPI solution (1 mg/mL) at 37°C for 30 min. After removing the staining solution, the apoptotic cells were visualized using fluorescence microscopy (Axiovert 200, ZEISS Inc., Germany and Olympus FV10i, Tokyo Inc., Japan).

### Detection of apoptosis

Apoptotic cells were visualized with an Annexin V-FITC/PI apoptosis detection kit (BD Biosciences, Inc) on a flow cytometer (GuavaReasyCyte flow cytometer, EMD Millipore, Billerica, MA, USA). Briefly, the ovarian cancer cells were seeded in 12-well plates and treated in a complete medium containing 0.1% DMSO and MHY2245 (0.03, 0.1, or 0.3 μM) for 48 h, followed by the harvesting and staining according to the manufacturer's protocol. The resulting images were collected and data were analyzed by Flowjo 7.6 software (Treestar, Ashland, OR, USA).

### Acridine orange assay

SKOV3 cells were seeded in 35 mm confocal dishes and treated with either MHY2245 (0.03, 0.1 and 0.3 μM) or DOX (1 μM) for 48 h, followed by staining with acridine orange (AO, 1 µg/mL) for 20 min at RT in a CO_2_ incubator. Subsequently, cells were washed twice with ice-cold PBS and observed under an inverted fluorescence phase contrast microscope (K1-Fluo, Nanoscope Systems®, Daejeon, Korea). Additionally, green (510-530 nm) and red (650 nm) fluorescence emission from blue (488 nm) excitation light was measured with a flow cytometer (Guava® easyCyte flow cytometer).

### Monodansylcadaverine (MDC) assay

Following incubation in confocal dishes, the cells were fixed with 4% paraformaldehyde in PBS at 10 min before being washed three times with PBS. These were treated with MDC (5 nmol/L; Sigma-Aldrich) for 30 min before viewing with a confocal laser scanning microscope (LSM 510, Carl Zeiss Co., Oberkochen, Germany).

### Lactate measurement

The cells pellets and medium were separated after drugs treatment for 48 h. These cells were then suspended in RPMI medium and centrifuged at 2000 xg for 5 min. The resulting cell pellets were resuspended in 500 µL extraction buffer containing internal standard (IS) thiamine at 4000 rpm for 5 min. A quantity of 100 µL of the supernatant was subjected to HPLC analysis using a C18 column (Synergi 4 µm Hydro-RP 80 Å, LC Column 250×4.6 mm) at a flow rate of 0.8 mL/min at room temperature. The mobile phase consisted of water with 0.1% phosphoric acid. DL-lactic acid was used as standards and for quantitative calibration in HPLC at 210 mm. Lactate (0.1-100 μg/mL; R2 = 0.997 ± 0.004) was detected by HPLC. Extraction recovery was found to be 85%, and the run time was 15 min. The linearity of each calibration curve was constructed by a weighted (1/x) linear regression method.

### Spheroid formation of SKOV3 cells

SKOV3 cells were collected in DMEM medium and harvested. The cell was seeded1000 cells per 100 μl medium each well in ULA round-bottom-96 plates (Corning, NY, USA). Plates are centrifuged (2000 rpm, 10 min) and incubated 3days for spheroid formed. After incubation, changed fresh medium (DMEM medium and supplemented with 10 % FBS and 1 % P/S) and treated MHY2245 (0.03, 0.1 and 0.3 μM) or DOX (1 μM) for 72 h. Spheroid images were taken collated using a the IncuCyte ZOOM system (Essen BioScience, MN, USA) and analyzed.

### In vivo tumor xenograft model

The animal experiments were approved by the Institutional Animal Care and Use Committee of Sungkyunkwan University (IACUC Number. SKKUIACUC2018-08-20-1). Female athymic nude mice (BALB-c nu/nu, 4-weeks old) were purchased from Japan SLC, Inc., (Hamamatsu, Shizuoka, Japan). The mice were maintained for 2 weeks in constant temperature (22 ± 2°C) and light (12 h light/dark cycle) conditions, in filtered-air laminar-flow cabinets. After 2 weeks acclimated, SKOV3 cells (1.5 × 10^7^) were suspended in 0.1 mL serum-free medium and 0.1 mL of Matrigel (BD Biosciences, Inc.) and injected subcutaneously (s.c.) into the left flank of the nude mice. Ovarian cancer cells are not easy to stick to the common nude mouse due to the specificity of the hormone 20. 17β-Estradiol (0.72 mM) was injected into all animals 3 times a week. Tumor sizes were checked daily until they reached ~200 mm^3^, and animals were then randomly divided into four groups (n=6 per group). MHY2245 (0.5 and 1 mg/kg) and DOX (1 mg/kg) were injected intraperitoneally (i.p.) in appropriate groups of animals once a week. Tumor sizes were measured using calipers and the tumor volumes were calculated using a standard formula (width x width x length x 0.52). After the final drug dose, all the mice were sacrificed and tumors were harvested for further analysis.

### Histological examination

A small part of the tumor tissues was embedded in paraffin wax after fixing in 10 % formalin for further analysis. Tissues were stained with hematoxylin and eosin (H&E) according to standard protocols. All histological images were captured under a fluorescence microscope.

### Immunohistochemical analysis

Formalin-fixed tumor tissues were embedded in paraffin and sectioned at 5 μm thickness. The sections were dewaxed in xylene and processed for antigen retrieval by placing them in 10 mM citrate buffer, and autoclaving for 20 min at 121°C. The sections were then incubated at 4℃ with primary antibodies for 24 h. For this, anti-Ki-67 (1:1000 diluted), anti-PKM2(1:1000 diluted), and anti-LC3B (1:200 diluted) antibodies were used as previously described. Antibody binding was visualized by using the ABC detection IHC kit (HRP/DAB, Abcam plc.). Samples were then incubated with diaminobenzidine and counterstained with hematoxylin. The images were captured using confocal microscopy. The quantification of the IHC images was performed using the IHC Profiler of Image-J software (NIH, Bethesda, MD). The IHC profiler was a system with automatic scoring of DAB colors. The DAB color grade was divided total 4 grade with high positive, positive, low positive and negative in pixel percentages.

### Statistical analysis

The data are expressed as the mean ± SEM of at least three independent experiments. Statistical analysis was performed using one-way analysis of variance (ANOVA) followed by Bonferroni's multiple comparison tests. *p <0.05; **p<0.01; and ***p<0.001 were considered statistically significant. All statistical comparisons were performed using *SigmaPlot* software and Statistical Package for the Social Sciences v.13 (SPSS, Inc., Chicago, IL, USA).

## Results

### Cytotoxicity of MHY2245 on various cancer or normal cell lines

**Figure 1:**
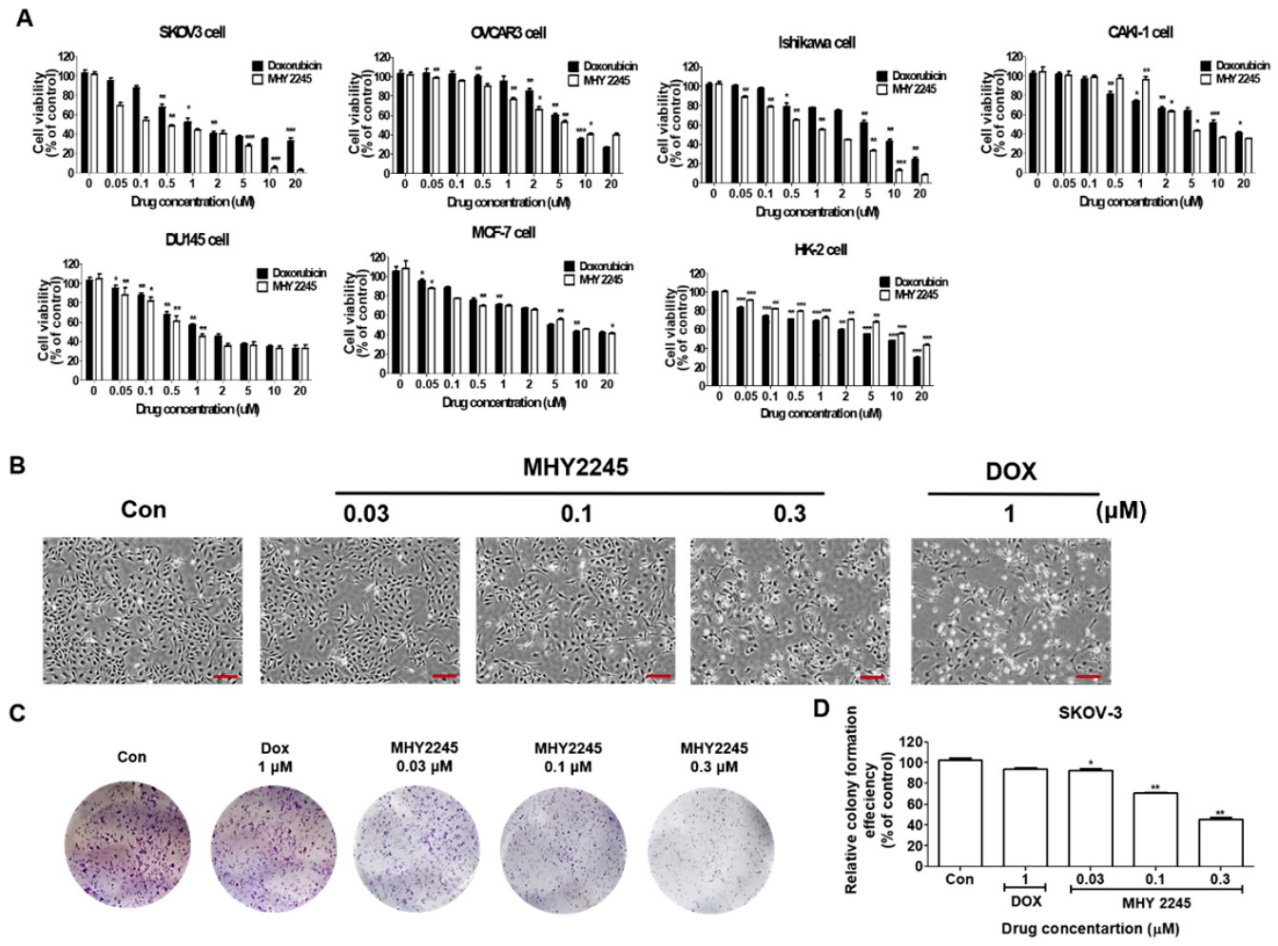
Cytotoxic effects of MHY2245 and doxorubicin (DOX) on human cancer or normal cell lines. (A) Cytotoxicity of MHY2245 and DOX against various human cancer or normal cell lines. Cells were treated with MHY2245 or DOX at various concentrations (0.01-20 µM) for 48 h. Cell viability was assessed using an MTT assay. Data represent the mean ± SD of three independent experiments. (B) Morphological changes (cell body shrinkage and reduction in cell number) in SKOV3 cells after MHY2245 (0.03, 0.1, or 0.3 µM) or DOX (1 µM) treatment for 48 h. Light microscopic images shown are representatives of three independent experiments. Scale bar: 100 µm. (C) Effects of MHY2245 or DOX on SKOV3 cell colony formation. Cells were treated with MHY2245 (0.03, 0.1 and 0.3 μM) or DOX (1μM) for 3 days. The fresh media changed every three days for 14 days. (D) Representative histogram showing the percentage of colony numbers. Data are expressed as mean ± SD of triplicate experiments. *p < 0.05, **p < 0.01 versus control group.

MHY2245 was synthesized in house (College of Pharmacy, Pusan National University, South Korea) using anthranilamide and 1-naphthaldehyde as previously described 21. We compared the cytotoxic effects of MHY2245 on six different human cancer cells including SKOV3 (human ovary cancer), OVCAR3 (human ovary cancer), Ishikawa (human endometrial cancer), DU145 (human prostate cancer), MCF-7 (human breast cancer) and Caki-1 (human kidney cancer) and normal cell lines (HK-2, human kidney normal tubular). MHY2245 was able to inhibit growth of human cancer cell lines in a dose-dependent manner, as observed after 48 h exposure (Figure 1A). MHY2245 exhibited potent cytotoxicity against all human cancer cell lines than that of doxorubicin (DOX; < 10 μM). The IC_50_ values of MHY2245 for SKOV3, OVCAR3, Ishikawa, DU145, Caki-1, and MCF-7 cells were 0.42, 6.35, 1.54, 0.78, 3.83, and 5.36 μM, respectively (Table 1), indicating that SKOV3 cells were particularly more sensitive to MHY2245 than the other cancer cell lines and the normal HK-2 cells (14.77 μM). Since SKOV3 cells showed the highest sensitivity to MHY2245, we selected them for further studies. The morphology of these cells under the microscope also suggested that MHY2245 is more toxic to SKOV3 cells (Figure 1B). We evaluated the long-term effect of MHY2245 on cell proliferation by ca colony formation assay, which is an effective method to determine the proliferation capacity of single cells. We evaluated the long-term effect of MHY2245 on cell proliferation by the colony formation assay, which is an effective method to determine the proliferation capacity of single cells. We found that MHY2245 more than DOX in OVCAR3 cells inhibited colony formation and DOX strongly inhibited colony formation in HK-2 cells (Supplementary Figure S1). Based on this, the MHY2245 effect of colony formation was verified in SKOV3 cells. As shown in Figure 1C and 1D, colony number was significantly reduced in SKOV3 cells after MHY2245 treatment compared to that of DOX.

### MHY2245 inhibits SIRT expression and HDAC activity

**Figure 2:**
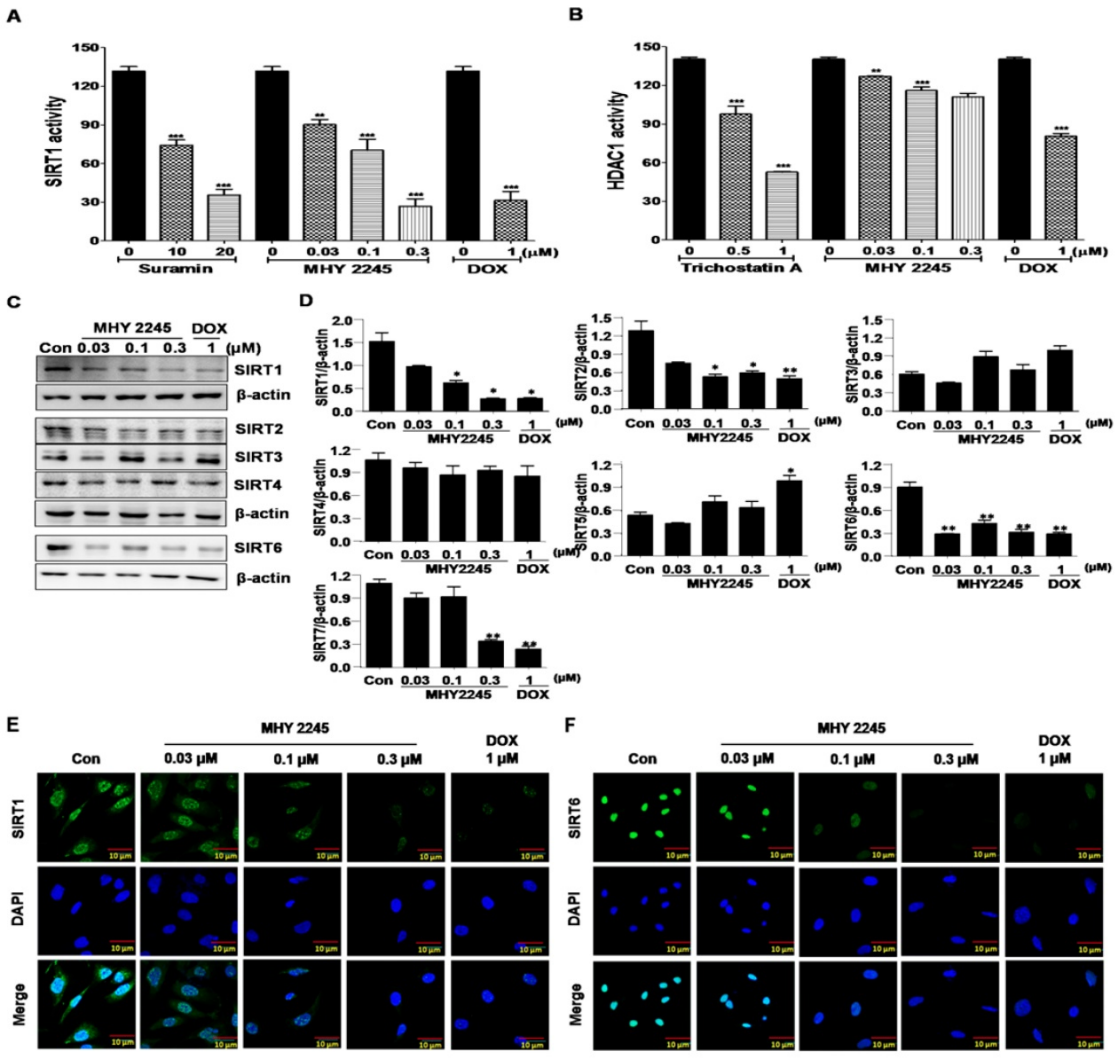
Effects of MHY2245 or doxorubicin (DOX) on SIRTs and HDAC1 enzyme activity. (A) SIRT1 and (B) HDAC1 enzyme activities were measured using a fluorogenic SIRT1 and HDAC1 assay kits. Results are expressed as mean ± SD of three independent experiments. Statistical analysis was done using ANOVA, followed by Bonferroni's multiple comparison tests. **p < 0.01 and ***p < 0.001 indicate significant differences between the vehicle controls and treatment groups. (C) Effects of MHY2245 and DOX on the expression of SIRT1, SIRT2, SIRT3, SIRT4, and SIRT6 proteins in SKOV3 cells. The cells were treated with indicated concentrations of MHY2245 and DOX for 48 h. β-Actin was used as a loading control. (D) A representative histogram showing the expression levels, which were quantified using Image J software compared to β-actin percentage in SKOV3 cells. Data are expressed as mean ± SD of triplicate experiments. *p < 0.05 and **p < 0.01 versus the control group. (E) Effects of MHY2245 and DOX on the SIRT1 protein levels in SKOV3 cells. SKOV3 cells were treated with indicated concentrations of MHY2245 or DOX for 48 h and monitored using fluorescence microscopy. (F) Effect of MHY2245 and DOX on the SIRT6 expression levels in SKOV3 cells. SKOV3 cells were exposed to the indicated concentrations of MHY2245 and DOX for 48 h and monitored using fluorescence microscopy. Immunofluorescence images were taken with a 50× magnification. Scale bar, 10 µm.

SIRT proteins play an important role in the survival and drug resistance of cancer cells. Typically, protein functions were associated with the repair of double-strand DNA breaks, cell-cycle progression and chromosome stability in biological functions and metabolism 22,23. The effects of MHY2245 on SIRT1 and HDAC1 enzyme activities were measured using an enzyme-linked immunosorbent assay (ELISA). As shown in Figure 2A, inhibition of SIRT1 enzyme activity by MHY2245 was more potent than suramin (a well-known SIRT1 inhibitor). Trichostatin A, a potent HDAC1 inhibitor, significantly inhibited HDAC1 enzyme activity, but MHY2245 is exerts a modest effect on HDAC1. The SIRT1, SIRT2, and SIRT6 protein levels were significantly reduced in SKOV3 cells after MHY2245 or DOX treatment (Figure 2C and 2D). Immunocytochemical (ICC) analysis showed that the nuclear expression of SIRT1 and SIRT6 proteins were dramatically reduced in SKOV3 cells by MHY2245 or DOX treatment (Figure 2E and 2F).

### MHY2245 induces cell cycle arrest at G2/M in SKOV3 cells

**Figure 3:**
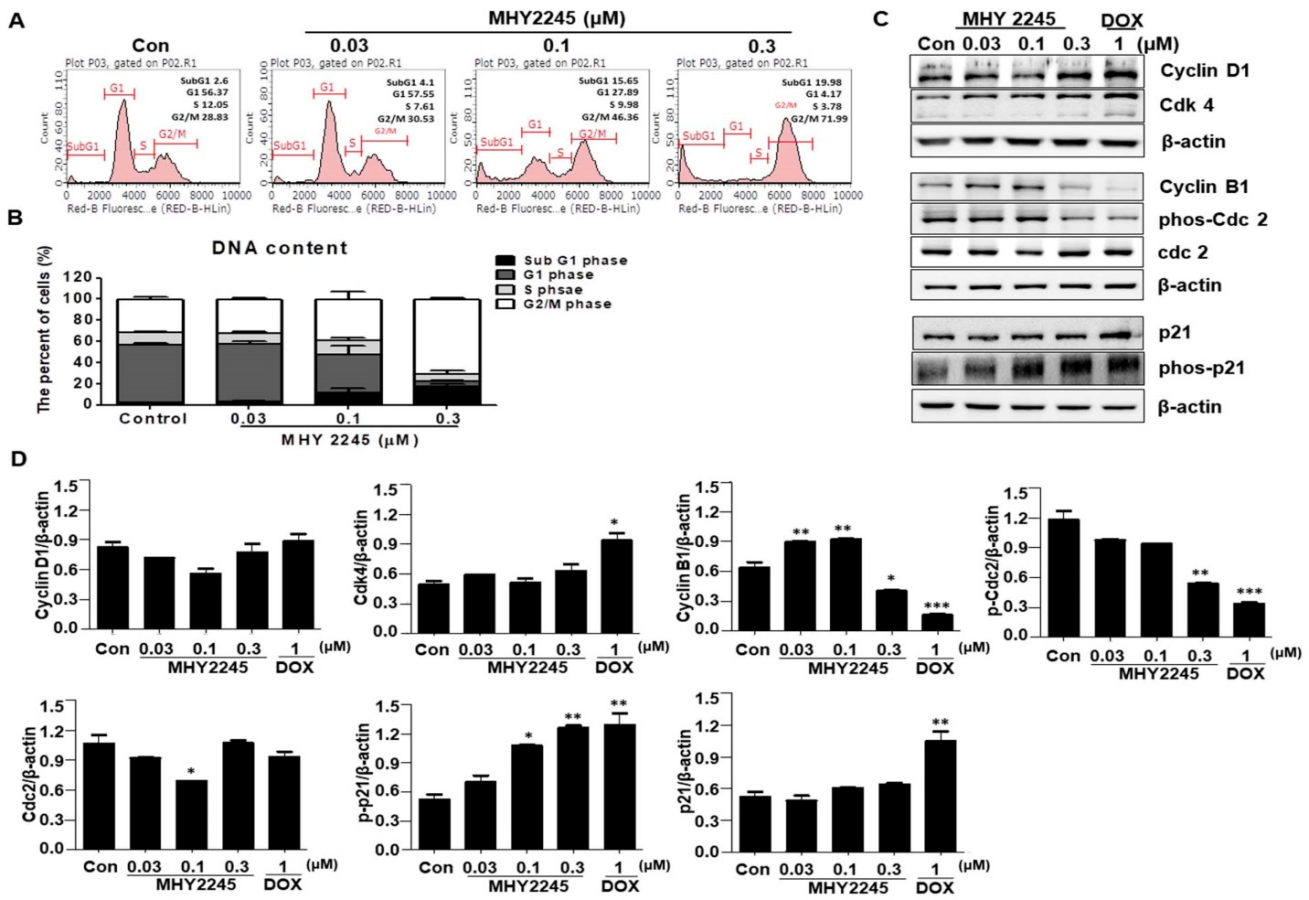
Effect of MHY2245 or doxorubicin (DOX) on cell cycle distribution in SKOV3 cells. (A) Cells were treated with MHY2245 or DOX at the indicated concentrations for 48 h, stained with propidium iodide (PI) and analyzed by flow cytometry to determine the cell cycle distribution. (B) Bar diagram showing the G1, S, and G2/M phase distributions of SKOV3 cells treated with vehicle control, MHY2245, or Doxorubicin (DOX). Data are expressed as mean ± SD of triplicate experiments. (C) Effect of MHY2245 or DOX on expression of the cell cycle regulatory proteins. SKOV3 cells were treated with MHY2245 (0.03, 0.1, or 0.3 µM) or DOX (1 µM) for 48 h. The expression levels of cyclin D1, Cdk4, cyclin B1, phospho-Cdc2 (p-Cdc2), Cdc2, p21, and phospho-p21 (p-p21) were detected by Western blot analysis. Data are representatives of three independent experiments. β-Actin was used as a loading control. (D) A representative histogram showing the expression levels, which were quantified using Image J software compared to β-actin percentage in SKOV3 cells. Data are expressed as mean ± SD of triplicate experiments. *p < 0.05 and **p < 0.01 and ***p < 0.01 versus the control group.

A previous study indicated that SIRT inhibitors induce G1/S or G2/M phase arrests and disrupt mitotic progression in various cancer cells 24,25. In this study, we found that MHY2245 increased the length of the G2/M phase in a concentration-dependent manner (Figure 3A**)**. Consequently, the proportion of G2/M phase cell populations increased up to 41.46% ± 2.32% upon MHY2245 0.3 μM treatment, as compared to control (29.3% ± 1.6%) (Figure 3B). Because these cancer cell growth inhibitions are useful for cancer treatment, we studied the regulatory protein of cell cycle using Western blot analysis. MHY2245 reduced the expression of cyclin B1 and phosphorylated Cdc2 (phos-Cdc2), as well as increased the expression of cyclin D1 and Cdk4, in SKOV3 cells (Figure 3C and 3D). In addition, MHY2245 significantly increased the level of phosphorylated p21 (phos-p21) expression.

### MHY2245 induces apoptosis in SKOV3 cells

**Figure 4:**
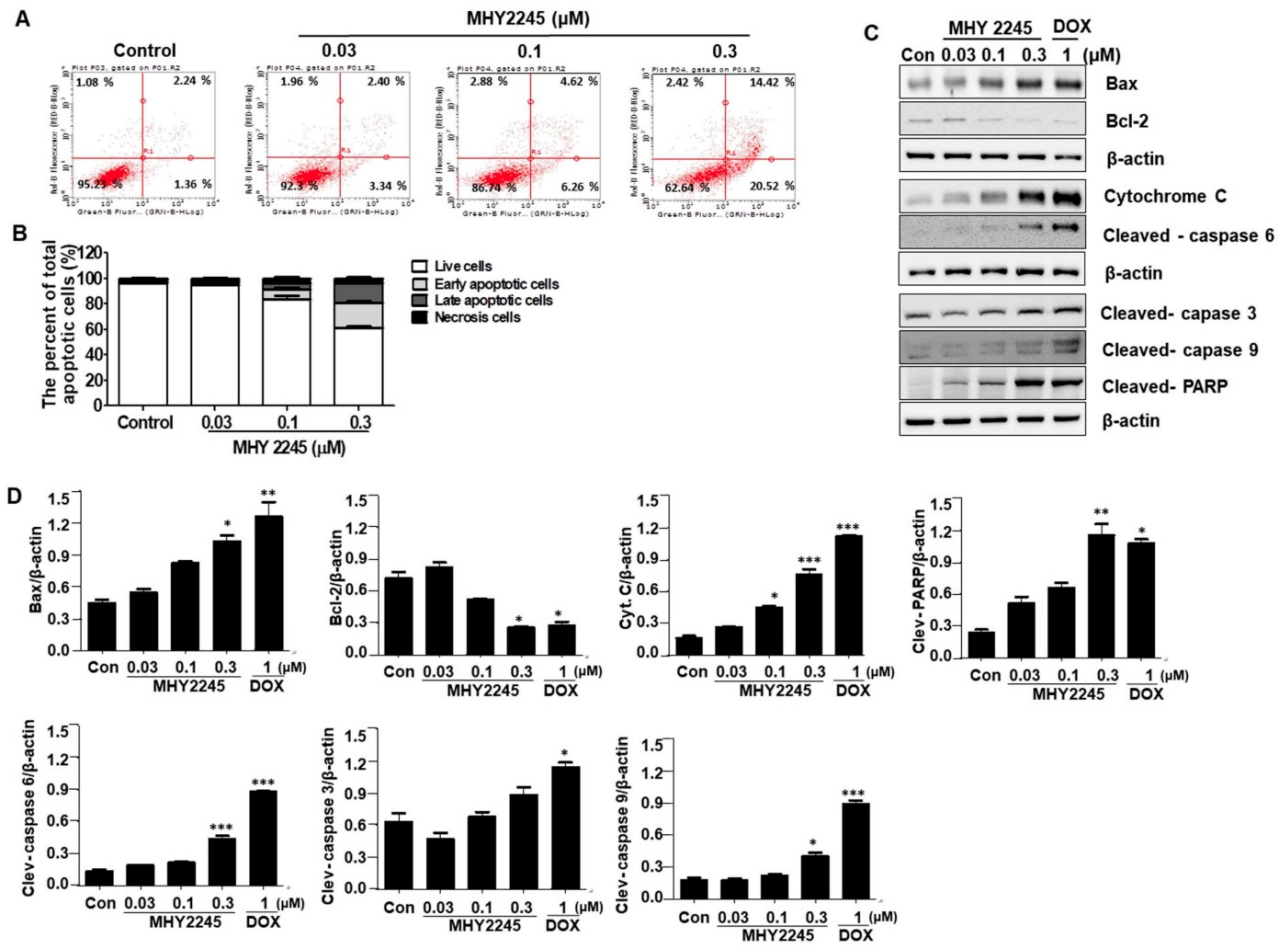
Effect of MHY2245 on apoptotic cell death pathways in SKOV3 cells. (A) Cells were treated with vehicle control, MHY2245, or doxorubicin (DOX) for 48 h. The flow cytometry profile represents AnnexinV-FITC staining on the *x* axis and PI staining on the *y* axis. (B) Bar graph showing the percentage of apoptotic cells. Data are expressed as mean ± SD of three experiments. (C) Cells were treated with vehicle control, MHY2245 (0.03, 0.1, or 0.3 µM) or DOX (1 µM) for 48 h and the expression of apoptosis-related proteins was analyzed by Western blot. β-Actin was used as a loading control. (D) Representative histogram showing the expression levels were quantified using Image J software compared to β-actin percentage in SKOV3 cells. Data are expressed as mean ± SD of triplicate experiments. *p < 0.05 and **p < 0.01 and ***p < 0.01versus the control group.

Three types of cell death usually occur in cancer cells, which include caspase-dependent apoptosis, necrosis, and autophagic cell death. In this study, we wanted to know which kind of pathways are involved in the death of ovarian cancer cells. To better understand the mechanisms of MHY2245 on anticancer activity, apoptotic cell death was determined using an Annexin V/PI binding assay, a DAPI staining, and Western blot analysis. We firstly performed a flow cytometric analysis to evaluate the early or late apoptotic cell populations after MHY2245 treatment. As shown in Figure 4A**,** apoptotic cells population was increased significantly by MHY2245 in SKOV3 cells; 5.78% (with 0.03 μM), 10.88% (with 0.1 μM) and 34.94% (with 0.3 μM), compared with the untreated control group (3.56%) (Figure 4B). Induction of apoptosis recognized as an efficient strategy for cancer chemotherapy and a useful indicator for successful cancer treatment and prevention 26. In view of the initial results, we extended the analysis to explore the effect of MHY2245 on expression of apoptosis-related molecular markers. Treatment with MHY2245 significantly increased the cleaved PARP, cleaved caspases, and Bax, cytochrome c in SKOV3 cells, while Bcl-2 levels decreased in a concentration-dependent manner (Figure 4C and 4D).

### MHY2245 induces autophagic cell death in SKOV3 cells

**Figure 5:**
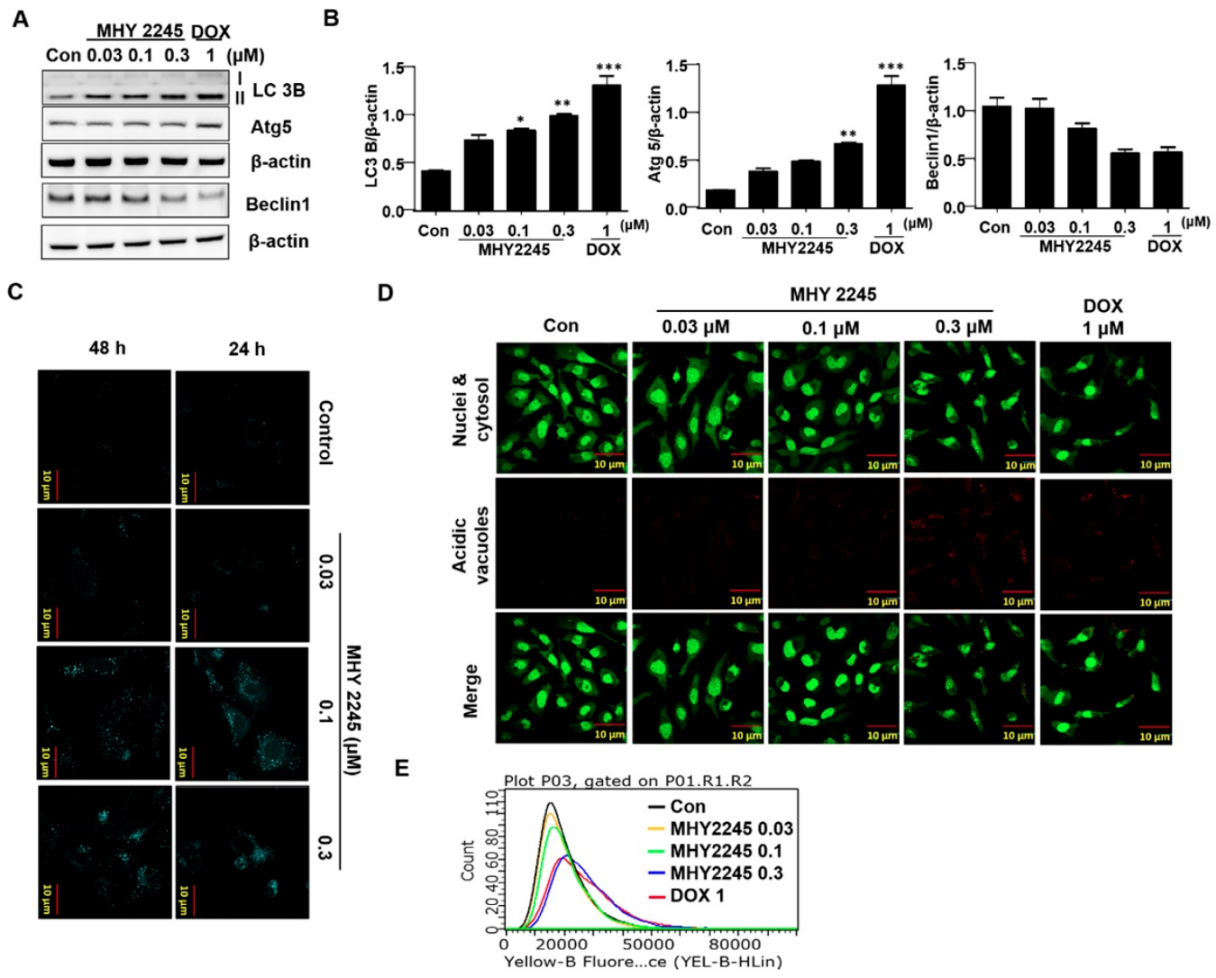
Effect of MHY2245 or doxorubicin (DOX) on the autophagic cell death pathway in SKOV3 cells. (A) Cells were treated with vehicle control, MHY2245 (0.03, 0.1 or 0.3 µM) or DOX (1 µM) for 48 h and the expression of autophagy-related proteins was analyzed by Western blot. β-Actin was used as a loading control. (B) Representative histogram showing the expression levels were quantified using Image J software compared to β-actin. Data expressed as mean ± SD of triplicate experiments. *p < 0.05 and **p < 0.01 and ***p < 0.01versus the control group. (C) Cells were treated with vehicle control, MHY2245 (0.03, 0.1 or 0.3 µM) or DOX (1 µM) for 48 h. After fixed, cells were incubated with MDC (0.05 mM) for 10 min at 37 °C and then washed four times with PBS pH 7.4. Cells were immediately analyzed by fluorescence microscopy using an confocal laser scanning microscope. (D) Acridine orange staining was used to detect the formation of autophagic vacuoles in SKOV3 cells treated with MHY2245 or DOX for 48 h. The cytoplasm and nucleolus fluoresce green, whereas the acidic compartments fluoresce bright red or orange-red colors. Images were observed using a confocal laser scanning microscope (LSM 510, Magnification × 400). Scale bar, 10 µm. (E) The acidic vacuoles were determined using flow cytometry.

Autophagy-related genes LC3-II and Atg5 may suppress tumor growth by inducing autophagy. Therefore, they are considered potential target proteins in cancer therapy. To evaluate the role of LC3-II in the regulation of autophagy cell death, we measured their expression by western blot analysis. We found that a significant increase in LC3-II levels were observed in SKOV3 cells treated with MHY2245 and Doxorubicin (Figure 5A). To determine the frequency of expression of the different types of autophagy-related proteins, beclin-1 and autophagy related proteins (Atg5) were measured. Atg5 is required for the formation of autophagosome in autophagy process 27. Similar to LC3-II levels, Atg5 levels were increased by MHY2245 and DOX treatment in SKOV3 cells, but an expression of beclin-1 levels were reduced (Figure 5A and 5B). To study whether autophagy is involved in MHY2245-induced cell death, we carried out MDC and acridine orange (AO) staining in SKOV3 cells. MDC and Acridine Orange (AO) staining have also been used to detect autophagic vacuoles 28. Similarly, NHY2245 induced apparent accumulation of MDC in the cytoplasmic vacuoles, whereas less accumulation of MDC was detected in control cells (Figure 5C). Similar results were also observed by AO staining. Numerous AO were observed in MHY2245-treated SKOV3 cells (Figure 5D and 5E). We found that MHY2245 significantly increased autophagy in a concentration-dependent manner. These observations suggest that the MHY2245-mediated autophagy contributes to overall cell death in SKOV3 cells.

### MHY2245 inhibits Akt/mTOR pathways and up-regulates acetyl p53 level

**Figure 6:**
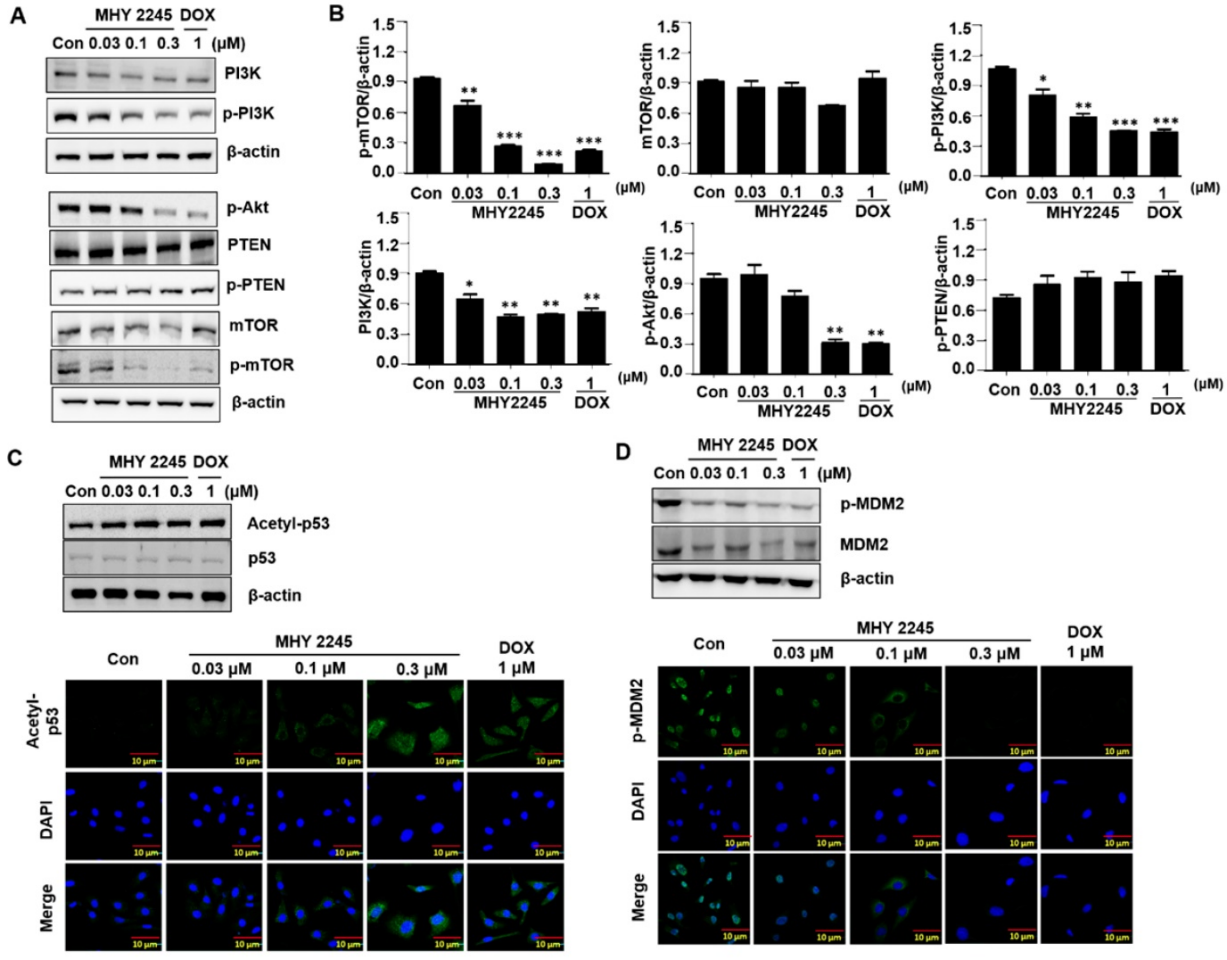
Effect of MHY2245 on Akt/mTOR pathways in SKOV3 cells. (A) Cells were treated with vehicle control, MHY2245 (0.03, 0.1 or 0.3 µM), or doxorubicin (DOX; 1 µM) for 48 h, and then the expression of PI3K, p-PI3K, Akt, p-Akt, PTEN, p-PTEN, mTOR, and p-mTOR levels were analyzed by Western blot. β-Actin was used as a loading control. (B) Representative histogram showing the expression levels were quantified using Image J software compared to β-actin. Data expressed as mean ± SD of triplicate experiments. *p < 0.05 and **p < 0.01 and ***p < 0.01versus the control group. (C) Cells were treated with vehicle control, MHY2245 (0.03, 0.1 or 0.3 µM) or DOX (1 µM) for 48 h and the acetylated p53 and p53 levels were analyzed by Western blot. β-Actin was used as a loading control (upper). SKOV3 cells were treated with MHY2245 or DOX for 48 h and fixed for immunostaining with antibodies against acetylated p53. Immunofluorescence and DAPI images were taken at a 50× magnification and merged (Lower). (D) Cells were treated with vehicle control, MHY2245 (0.03, 0.1 or 0.3 µM) or DOX (1 µM) for 48 h and the MDM2 and p-MDM2 expression was analyzed by Western blot. β-Actin was used as a loading control (upper). SKOV3 cells were treated with MHY2245 or DOX for 48 h and fixed for immunostaining with antibodies against p-MDM2. Immunofluorescence images were taken with a 50× magnification (lower). Scale bar, 10 µm.

Previous studies have indicated that a typical anticancer treatment inhibited AKT/mTOR/PI3K pathways, in addition to cell death in cancer cell lines 29. To further understand the anticancer mechanism of MHY2245, we examined its effects on the AKT/mTOR/PI3K and p53 signaling pathway. We found that phospho-Akt, phospho-mTOR and phospho-PI3K were down-regulated by MHY2245 in SKOV3 cells (Figure 6A and 6B). Studies show that apoptosis is accompanied by increased p53 expression 30,31. Furthermore, SIRT inhibitors exert their anticancer effects by inducing the acetylation of histones in cancer cells 31. Using Western blot and immunocytochemistry, we found that MHY2245 significantly increased p53 acetylation (acetyl-p53) and inhibited phosphorylated-MDM2 (p-MDM2) expression (Figure 6C and 6D) in SKOV3 cells.

### MHY2245 regulates reprograming of cancer cell metabolism

**Figure 7:**
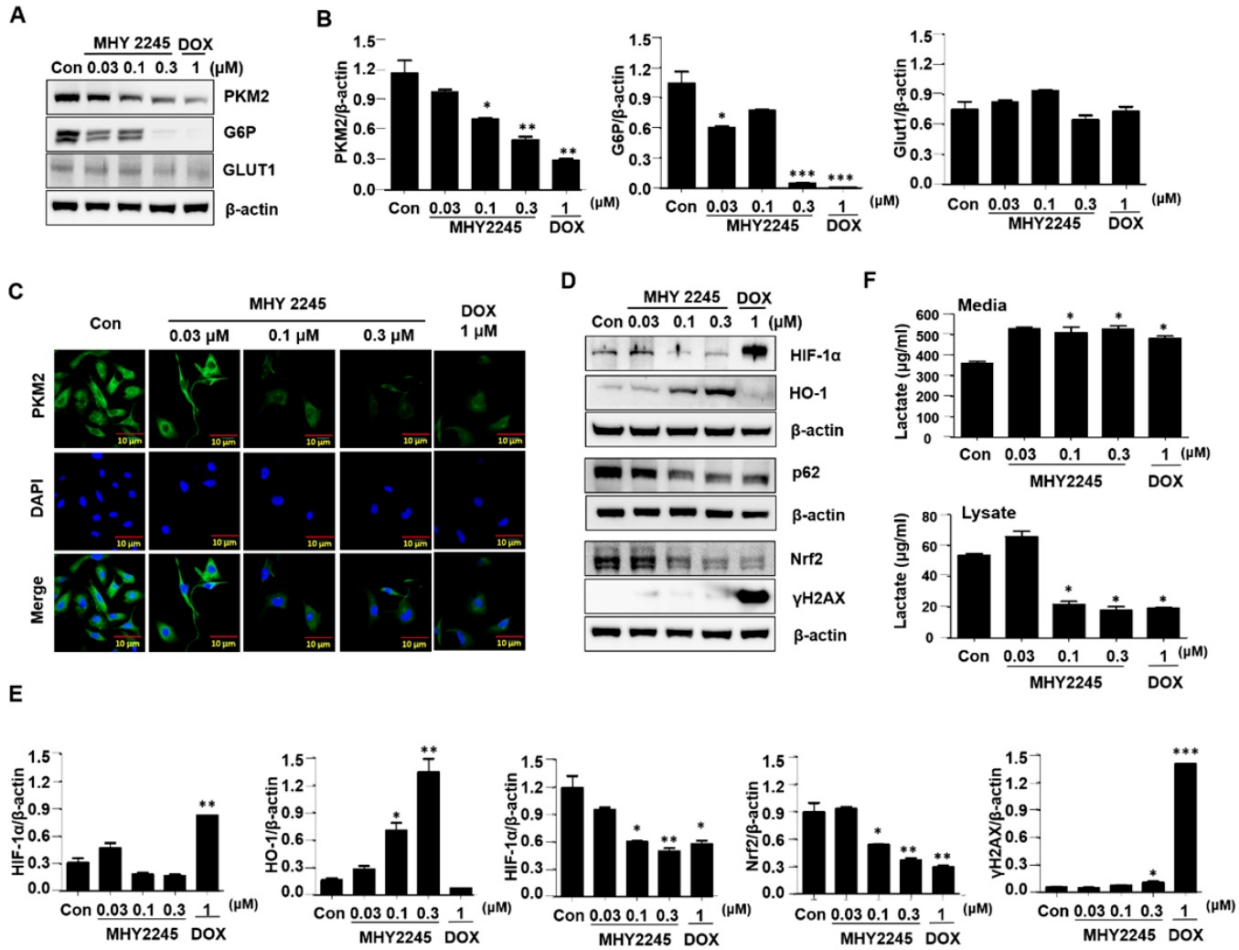
Effect of MHY2245 on energy metabolism in SKOV3 cells. (A) Cells were treated with vehicle control, MHY2245 (0.03, 0.1 or 0.3 µM) or doxorubicin (DOX; 1 µM) for 48 h and the expression of PKM2, G6P, and GLUT1 levels was analyzed by Western blot. β-Actin was used as a loading control. (B) A representative histogram showing the expression levels, which were quantified using Image J software compared to β-actin. Data expressed as mean ± SD of triplicate experiments. *p < 0.05 and **p < 0.01 and ***p < 0.01 versus the control group. (C) Cells were treated with vehicle control, MHY2245 (0.03, 0.1 or 0.3 µM), or DOX (1 µM) for 48 h andPKM2 levels (Green) were determined by immunofluorescent staining. The nuclei (blue) were stained with DAPI. Immunofluorescence images were taken with a 50× magnification. Scale bar, 10 µm. (D) Cells were treated with vehicle control, MHY2245 (0.03, 0.1 or 0.3 µM) or DOX (1 µM) for 48 h and the expression of HIF-1α, HO-1, p62, Nrf-2, and yH2AX levels was analyzed by Western blot. β-Actin was used as a loading control. (E) A representative histogram showing the expression levels, which were quantified using Image J software compared to β-actin percentage. Data are expressed as mean ± SD of triplicate experiments. *p < 0.05 and **p < 0.01 and ***p < 0.01 versus the control group. (F) Effects of lactate production by MHY2245 or DOX in SKOV3 cells. Cells were treated with vehicle control, MHY2245 (0.03, 0.1 or 0.3 µM) or DOX (1 µM) for 48 h and lactate concentration of the cellular or media were analyzed according to described in Materials and Methods. Results are expressed as mean ± SD of triplicate experiments. *p < 0.05 versus the control group.

To identify the mechanisms through which MHY2245 affected aerobic glycolysis, we evaluated the expression pattern of PKM2 in the cancer cells treated with MHY2245 or DOX. Immunoblotting showed that MHY2245 significantly reduced the PKM2 protein level in SKOV3 cells in a dose-dependent manner (Figure 7A and 7B). This observation was confirmed through immunofluorescence staining (Figure 7C). We also evaluated the glucose uptake and lactate formation in SKOV3 cells treated with MHY2245 because up-regulation of glycolysis and PKM2 levels has been observed in many types of cancer cells, it is reasonable to assume that down-regulation of PKM2 inhibits glycolysis and thereby blocks the proliferation of cancer cells. MHY2245 significantly down-regulated the glucose-uptake-related proteins G6P and GLUT1 levels (Figure 7B). These results were confirmed by immunofluorescence staining of SKOV3 cells counterstained with DAPI. As shown in Figure 7C, cytoplasmic and nuclear levels of PKM2 were reduced in SKOV3 cells treated with MHY2245. Further, to elucidate the mechanism responsible for PKM2 down-regulation, we measured the levels of hypoxia-inducible factor 1-alpha (HIF1α), heme oxygenase-1 (HO-1), p62, and nuclear factor erythroid 2-related factor 2 (Nrf-2) 32, 33. HIF-1α and Nrf2 protein levels were significantly reduced in SKOV3 cells after MHY2245 treatment. However, the level of the DNA damage marker γH2AX did not change by MHY2245 treatment (Figures 7D and 7E). Furthermore, the cellular concentration of lactate was significantly reduced by MHY2245 treatment in the lysate of SKOV3 cells (Figure 7F).

### MHY2245 inhibits human ovarian cell tumors in xenograft models

**Figure 8:**
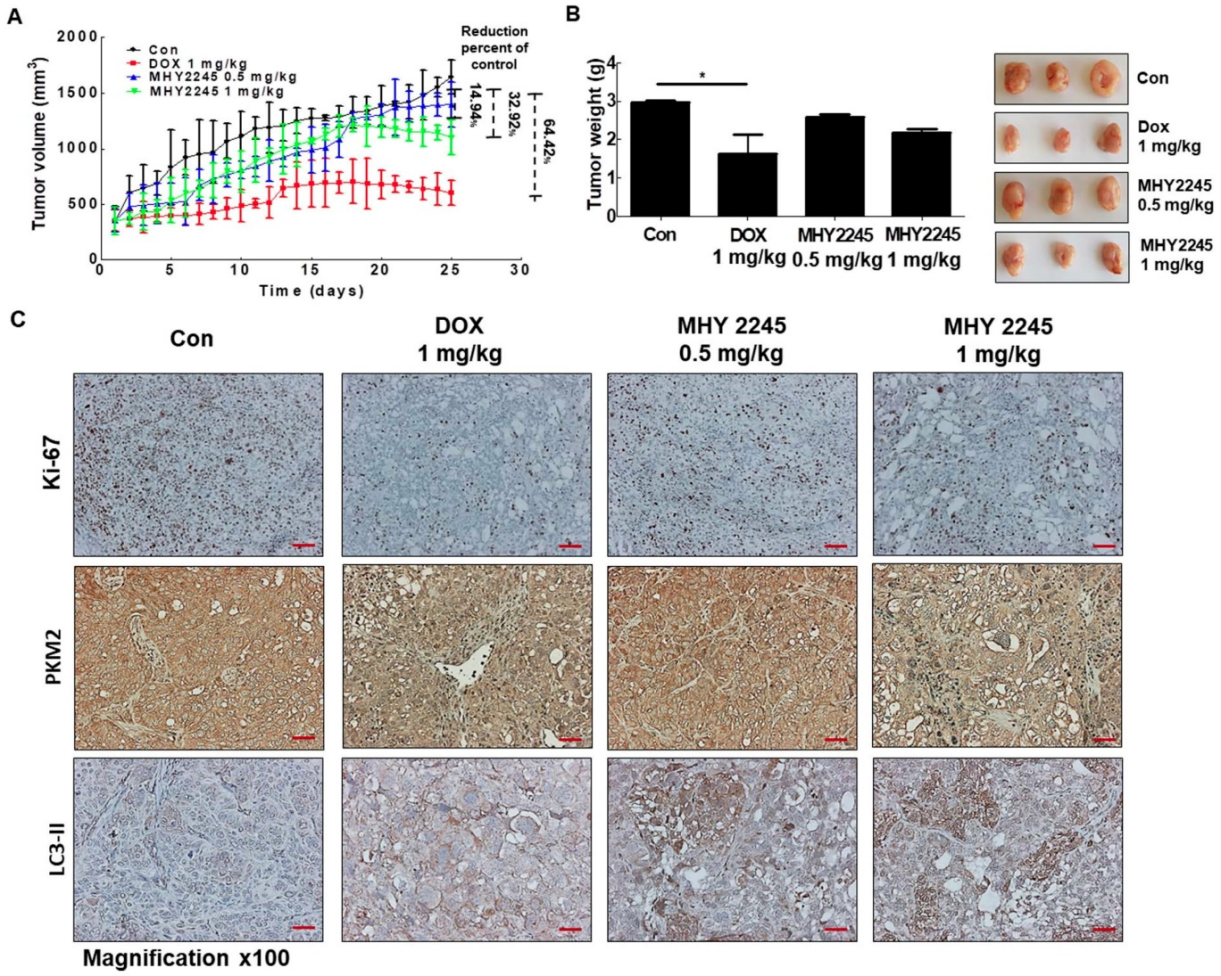
** Anticancer effect of MHY2245 in *in vivo* xenograft model mice.** The SKOV3 cells were injected into the dorsal subcutaneous skin of nude mice. On the fourteenth day, MHY2245 (0.5 and 1 mg/kg/day) or doxorubicin (DOX; 1 mg/kg/day) were intraperitoneally injected into the mice for 4 weeks. (A) Tumor volume was measured twice weekly, as described in Materials and Methods. Results are indicated as mean ± SD of six animals. (B)Animals were sacrificed, and tumor tissues were collected at the end of treatment duration, and tumors were weighed. Tumor weights are indicated as mean ± SD of six animals. *p < 0.05versus the control group. (C) The tumor tissues were stained with the Ki-67, PKM2, and LC3-II antibodies and examined under ×100 magnification.

A previous study indicated, 3D multicellular spheroid-based assays have been used for studying tumor growth 34,35. We measured the potency of MHY2245 or DOX to inhibit the growth of SKOV3 cells in 3D spheroidal models. Results showed that after treated with MHY2245 significantly reduced the diameter spheroids in a concentration-dependent manner (Supplementary Figure S2). To further investigate the anti-tumor activity of the MHY2245 in xenograft model, we subcutaneously injected SKOV3 cells into 6-week-old female athymic nude mice. We found that all these mice developed tumors in one week. As shown in Figure 8A and 8B, tumor size and tumor weight were significantly reduced in the MHY2245-treated group compared with those in the control. IHC staining for the widely used proliferation marker Ki-67 showed that MHY2245 treatment markedly reduced the number of proliferative cells relative to that in the untreated control tumors (Figure 8C, upper). In addition, we observed by expressing of LC3-II and PKM2 in SKOV3 tumor. As a result, increased in expression of LC3-II and inhibited of PKM2 expression was exhibited in SKOV3 tumor treated with MHY2245 (Figure 8C, middle and lower).

## Discussion

SIRT1 is plays an important role in cell survival, signal transduction, and apoptosis, by deacetylation of specific cell signaling molecules (p53, FOXO3, FOXO4, NF-kB, and HIFs) 36,37. However, a few studies have suggested that SIRT1 may act as a tumor activator in various human cancers, including breast cancer, prostate cancer, or ovarian cancer 38,39. Indeed, the expression of SIRT1 was significantly different according to tumors type. Previous study indicated that the expression of the SIRT1 level was markedly lower in the tumor tissues of breast cancer, prostate cancer, bladder cancer, ovarian cancer, and glioblastoma as compared to normal tissues 39,40. Conversely, over-expression of SIRT1 was frequently observed in all kinds of non-melanoma skin cancers, including squamous cell carcinoma, basal cell carcinoma, Bowen's disease, and actinic keratosis 41,42. Additionally, expression of SIRT1 was more strongly expressed in OVCA3 cells than in immortalized ovarian surface epithelium OSE7E cells 43. In particular, over-expression of SIRT1 is correlated with lymph node metastasis and the decrease of the 5-year survival rate, indicating its oncogenic effects in ovarian cancer 40,42. Furthermore, overexpression of SIRT1 increased the chemotherapy resistance in IGROV1 cells and knock-down of SIRT1 led to cell growth arrest and enhanced the sensitivity of drug-resistant cells to DOX 44. Therefore, inhibition of SIRT1 resulted in anticancer effects in SKOV3 cells through apoptotic or autophagic cell death pathways 45. SIRT1 enhanced cell proliferation, chemo-resistance and aggressiveness by up-regulating multiple antioxidant pathways to inhibit oxidative stress 46. These differences can be influenced by the tumor types and tumor microenvironment, as well as other factors which are regulated by SIRT-mediated target gene expression. Here, we investigated the functional role of the novel SIRT1 inhibitor MHY2245 on the regulation of apoptosis and autophagic cell death in ovarian cancer cells, as well as its associated mechanisms.

In this study, the anticancer effects of MHY2245 were evaluated against six different human cancer and normal (HK-2 cells) cell lines. The cytotoxicity of MHY2245 was significantly higher in SKOV3 cells than in human normal kidney HK-2 cells. Among cancer cell lines, SKOV3 cells showed the highest sensitivity to MHY2245-induced cytotoxicity than other cancer cell lines. SIRT1 enzyme activity was also reduced by MHY2245 treatment in a dose-dependent manner. Among all the SIRT isoforms, expression levels of SIRT1, SIRT2, and SIRT3 were significantly reduced in SKOV3 cells by MHY2245 treatment and may contribute to the inhibition of SKOV3 cells' proliferation. These results were supported by the visually altered morphological changes in SKOV3 cells, suggesting that MHY2245 showed a selective cytotoxicity to human ovarian cancer cells. We performed soft-agar colony formation assays to further examine the effects of MHY2245 on SIRT1-mediated SKOV3 cell proliferation. Finally, inhibition of SIRT1 by MHY2245 treatment significantly reduced colony formation abilities as compared to the controls. These results suggest that SIRT1 enhanced the tumor formation ability and increased the expression of cell-cycle-progression-associated genes in SKOV3 cells.

We were unable to demonstrate the effects of SIRT1 on well-known survival and apoptotic factors, including p-Akt, Bcl-2, or Bax. We examined the apoptosis-inducing potency of MHY2245, using the SKOV3 ovarian carcinoma cell line because this cell line demonstrates a substantial expression level of SIRT1, has mainly null-typep53 genes and this type of cancer is the most prevalent malignancy in women. Therefore, we performed apoptotic assays with Annexin V/PI staining methods to evaluate the percentage of apoptotic cells. Our data indicated that MHY2245 significantly increased the number of apoptotic cells in SKOV3 cells, as seen in the flow cytometric assay. On the other hand, although MHY2245 can effectively induce inhibition of SIRT1 and subsequent cancer cell death, it cannot do so in p53-null cancer cells. These results revealed that the apoptotic sensitivity of SKOV3 cells to MHY2245 was negligible. This is in accordance with findings of Lara and coworkers 47, who showed that SIRT1inhibitors can induce massive apoptosis in p53-dependentcancers, but not in p53-independent cells. Our previous study showed the strongest induction of apoptosis in MCF-7 cells after treatment with salermide, a SIRT1 inhibitor, but SKOV3 cells showed only about a 10% increase in apoptosis 45,48-50. It appears that this observation in MCF-7 cells was due to the presence of the wild-type p53, since p53-deficient SKOV3 cells show only a small increase in apoptosis due to salermide 51. p53 is an important target protein for SIRT1.Previous study indicated that the selective SIRT1 inhibitor, EX527, significantly suppressed the proliferation of endometrial carcinoma cells regardless of the p53 mutation status 52,53. Thus, activation of the p53 pathway through SIRT1 inhibition has been shown to have powerful anticancer effects 54. Our present data indicate that activation of the apoptosis-related signal pathway via up-regulation of p53, plays a pivotal role in MHY2245-induced cell death. Thus, a novel SIRT inhibitor may be a useful agent for the treatment of malignancy, especially with over-expression of SIRTs.

Autophagy, another important dynamic process in cell death, is characterized by degradation of cytoplasmic components such as mitochondria, through complicated intracellular reorganization with lysosomes 55,56. To investigate the possible association between SIRT1 inhibition and autophagic cell death, we measured the autophagy-associated proteins LC3-II and beclin-1 in SKOV3 cells. LC3-II expression had increased in SKOV3 cells which are p53-null, suggesting an association of SIRT1 inhibition with expression of LC3-II. Previously, sirtinol, a potent SIRT1 inhibitor, was shown to induce autophagic cell death in MCF-7 cells 57. Likewise, MHY2245 markedly increased LC3-II levels and autophagy-related proteins such as Atg3 and Atg5 in SKOV3 cells. Therefore, p53 had no effect on the physical interaction between beclin-1 and Bcl-2, indicating that MHY2245-induced autophagic cell death may be independent of the p53-related pathway. However, we found that Bax levels were substantially increased by MHY2245 treatment in p53-null SKOV3 cells, and so, we assumed that Bax might be involved in the increase of autophagy protein levels in the presence of MHY2245. Based on autophagy in p53-null SKOV3 cells, we conclude that increased autophagy induction by MHY2245 contributes to increased apoptosis only in the presence of active p53.

It is vital to recognize that SIRT1 promotes cancer progression through modulating Akt activity 49,58. However, such molecular mechanisms underlying the progress of tumor by SIRT1 have not been reported. As a result, controlling the expression of SIRT1 might be an alternative approach to manage the PI3K/Akt/mTOR pathway in ovarian cancer cells. PI3K/Akt/mTOR pathways are a key player in various types of malignant human tumors, such as breast cancer, lung cancer, melanoma, and lymphoma. These pathways are significantly activated during tumor development and involved in various related processes including cancer cell adhesion, growth, migration, invasion, and angiogenesis 59,60. Recent literature suggested that p53 is deacetylated by SIRT1, which leads to increased p53 function 61,62. We showed that down-regulation of SIRT1 caused a significant reduction in phosphorylated Akt expression, and SIRT1 directly interacted with Akt in ovarian cancer cells. Therefore, our data suggest that MHY2245 inhibited ovarian cancer cell proliferation and induced autophagy by inhibiting the SIRT1/Akt/mTOR pathway. These results revealed that intricate connections in the SIRT1/Akt signaling axis could significantly affect the proliferation of ovarian cancer. In addition, the mTOR/AMPK pathway plays a critical role in the regulation of the ovarian cell proliferation, apoptosis, autophagy, and cell metabolism 63,64. Therefore, inhibiting Akt/mTOR, as well as inhibiting PKM2, by MHY2245 treatment can increase autophagy, which may enhance antitumor effects of MHY2245 in ovarian cancer cells.

The anti-tumorigenic effects of MHY2245 were also demonstrated using an in vivo xenograft model, in which the SKOV-3 cell tumor was significantly reduced by MHY2245. A significant decrease in PKM2 levels was observed in MHY2245-treated tumor tissues, as compared to control. These results suggest that MHY2245-mediated PKM2 inhibition is closely related to autophagic cell death in SKOV3 cells via changes in cancer cell metabolism. Taken together, our data suggest that inhibition of SIRT1 by MHY2245 causes inhibition of cancer cell metabolism by decreasing PKM2 expression. These findings indicate that MHY2245 promotes apoptosis and autophagy in ovarian cancer cells via PI3K/Akt/mTOR-mediated pathways.

In conclusion, this study is the first to evaluate the anticancer activity of MHY2245 on human ovarian cancer cells. It inhibits cell proliferation and induces autophagic cell death by inhibiting PKM2-mediated cancer cell metabolism via the PI3K/Akt/mTOR pathway. In this study, we suggest that a synthetic SIRT inhibitor represents a potential for ovarian cancer therapy that has a strong inhibitory activity on SIRT1. Our research provides new targets for MHY2245 for ovarian cancer treatment.

## Supplementary Material

Supplementary figures.

## Figures and Tables

**Table 1:** The IC_50_ values are indicated mean ± SD of 3 independent experiments.

Cell lines	IC_50_ values (μM)	
	MHY2245	DOX
SKOV3	0.42± 0.07	1.38± 0.11
MCF-7)	5.36± 0.2	7.92± 0.32
Ishikawa	1.54± 0.09	8.16± 0.25
DU145	0.78± 0.11	1.5± 0.27
CAKI-1	3.83± 0.3	9.67± 0.74
OVCAR3	6.35± 0.81	6.96± 0.65
HK-2	14.77±1.21	7.45± 1.19
