# Supplementary material for: A new SIRT1 inhibitor, MHY2245, induces autophagy and inhibits energy metabolism via PKM2/mTOR pathway in human ovarian cancer cells
Source: Int J Biol Sci. 2020 Apr 6;16(11):1901–16. doi: 10.7150/ijbs.44343 (PMC7211172; doi:10.7150/ijbs.44343)
Supplement: Supplementary file 1 — Supplementary figures. [file ijbsv16p1901s1.pdf]

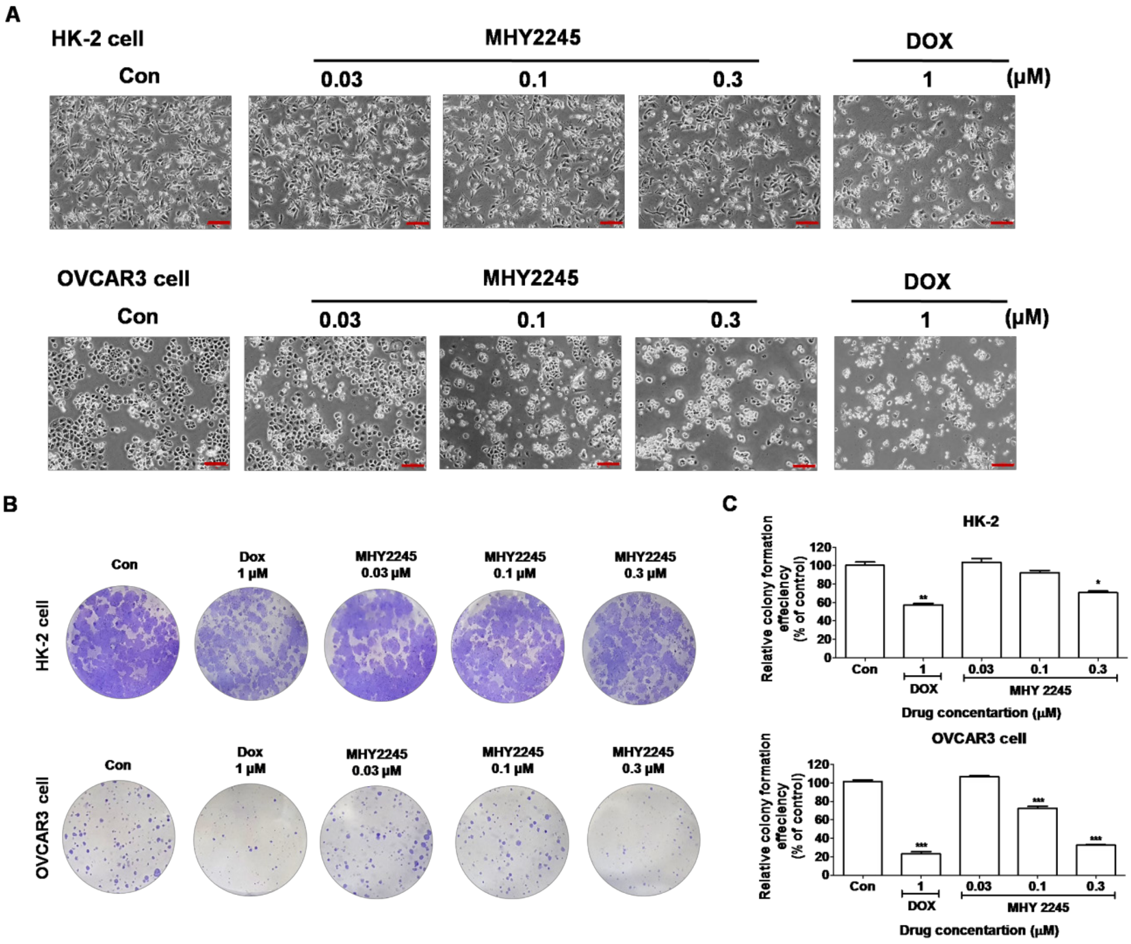

Supplementary Figure 1. Effect of MHY2245 and doxorubicin (DOX) on colony formation in human ovarian cancer or normal kidney cells. (A) Morphological changes (cell body shrinkage and reduction in cell number) in HK-2 (upper) and OVCAR3 (lower) cells after MHY2245 (0.03, 0.1, or 0.3 μM) or DOX (1 μM) treatment for 48 h. Light microscopic images shown are representatives of three independent experiments. Scale bar, 100 μm. (B) Effects of MHY2245 or DOX on HK-2 (upper) and OVCAR3 (lower) cells colony formation. MHY2245 (0.03, 0.1 and 0.3 μM) or DOX (1μM) treatment for 24 h after colony formed. All drug treatment performed every three days for 14 days. (C) Representative histogram showing the percentage of colony numbers. Data are expressed as mean ± SD of triplicate experiments. *$p < 0.05$, **$p < 0.01$ versus control group.

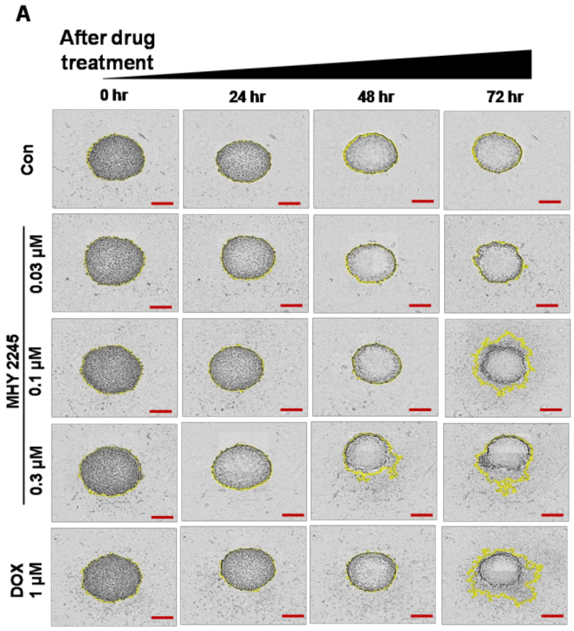

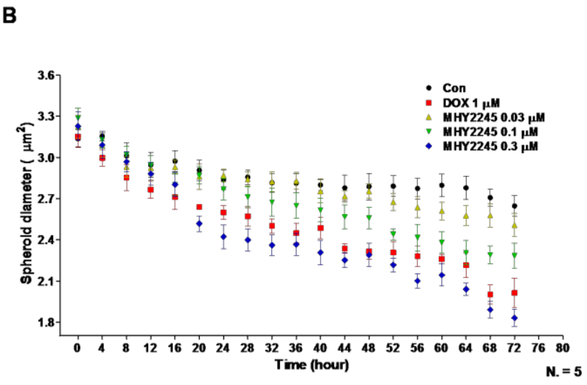

	Spheroid diameter (%)				
	Con	DOX 1 µM	MHY2245 0.03 µM	MHY2245 0.1 µM	MHY2245 0.3 µM
0 hr	100 % (3.13 µm²)	100 % (3.15 µm²)	100 % (3.22 µm²)	100 % (3.28 µm²)	100 % (3.23 µm²)
24 hr	90.44 % (2.83 µm²)	82.47 % (2.60 µm²)	88.99 % (2.87 µm²)	84.24 % (2.76 µm²)	75.04 % (2.42 µm²)
48 hr	88.85 % (2.78 µm²)	73.48 % (2.31 µm²)	85.39 % (2.75 µm²)	77.84 % (2.55 µm²)	70.91 % (2.29 µm²)
72 hr	84.37 % (2.64 µm²)	63.86 % (2.01 µm²)	77.76 % (2.50 µm²)	69.48 % (2.28 µm²)	56.72 % (1.83 µm²)

Figure S2. Anti-tumor potency of MHY2245 or doxorubicin (DOX) in 3D spheroid assay model of SKOV3 cells. SKOV3 cells were incubated for 3 days after seeding cells (1000 cells/well) in round-bottom plates. After, treated MHY2245 (0.03, 0.1 and 0.3 μM) or DOX (1μM) for 72 h. (A) Morphology of SKOV3 spheroids with different concentrations from day 1 to day 3. Images were taken with a 4× magnification using an IncuCyte ZOOM system (Essen BioScience, MN, USA). Scale bar, 500 μm. (B) The diameter of spheroids with different concentrations of drugs as a function of culturing time in 96-well round-bottomed plates. Data represents mean ± S.D. from 2 independent measurements, each in duplicate (n = 5).
